# Cyclosporin-Induced Gingival Enlargement in a Periodontitis Patient With Pemphigus Vulgaris: A Case Report

**DOI:** 10.1155/crid/8318894

**Published:** 2025-02-11

**Authors:** Wee-Jian Chong, Norliwati Ibrahim, Shahida Mohd-Said

**Affiliations:** ^1^Department of Restorative Dentistry, Universiti Kebangsaan Malaysia, Kuala Lumpur, Malaysia; ^2^Department of Craniofacial Diagnostics & Biosciences, Universiti Kebangsaan Malaysia, Kuala Lumpur, Malaysia

**Keywords:** chronic oral ulcerations, desquamative gingivitis, drug-influenced gingival enlargement, intraepithelial bullae, pemphigus vulgaris, vesiculobullous lesions

## Abstract

**Background:** Pemphigus vulgaris (PV) is a chronic autoimmune disorder affecting mucous membranes and skin, with potential life-threatening risks. It is typically characterized by blisters within the oral cavity with or without subsequent skin involvement. Given the importance of timely intervention, dental professionals are responsible for diagnosing this condition, as prompt detection and intervention greatly influence the disease progression and prognosis.

**Case Description:** A 44-year-old male patient presented with swollen and bleeding gums, accompanied by multiple chronic ulcers in the oral cavity. He was initially diagnosed with PV in 2018; his case posed significant challenges, including drug-influenced gingival enlargement and the psychological burden of managing a chronic, relapsing condition.

**Management and Prognosis:** The patient received treatment with an immunosuppressive medication (cyclosporin) along with long-term systemic steroids (prednisolone). In November 2022, cyclosporin was replaced with a steroid-sparing medication (methotrexate) to control drug-influenced gingival enlargement. The periodontal condition improved after 3 months of changing the medication regimen, nonsurgical periodontal therapy, and reinforced oral hygiene practices. The patient undergoes regular medical evaluations every 6 months with the dermatology department.

**Clinical Implications:** Effective management of PV necessitates long-term systemic steroid therapy, often supplemented with immunosuppressive agents, to control the disease and minimize relapse risks. Regular clinical assessments are essential for patients receiving steroid and immunosuppressive treatment to monitor potential side effects, including cyclosporin-induced gingival enlargement. If gingival enlargement is compounded by periodontal disease, it can further complicate the management of PV. Drug-induced gingival enlargement has a favorable prognosis and is reversible upon discontinuation or substitution of the causative medication. An interdisciplinary approach involving primary clinicians, dentists, and the healthcare team is crucial to addressing the patient's signs and symptoms effectively.

## 1. Introduction

Pemphigus encompasses a group of autoimmune diseases characterized by forming vesicles or bullae within the intraepithelial region of skin and mucous membranes [1]. Pemphigus vulgaris (PV) is the most prevalent subtype of pemphigus, comprising up to 70% of all cases [2]. The mortality rate of PV has decreased in recent years due to therapeutic advances [3], but it is still an important cause of significant mortality [3]. PV treatment has been a challenge during the past decades and continues to be so [4], especially in treating oral PV, as the first site of involvement in two-thirds of PV patients is the oral mucosa [5]. Oral PV is usually described as desquamative gingivitis and/or as vesiculobullous lesions of the free and attached gingiva characterized by intraepithelial bullae that, after rupture, leave erosions [6]. Oral PV is mostly recalcitrant and challenging due to the chronic nature of the disease and the rough oral environment [7]. Although PV is indirectly caused by dental biofilm, its clinical course may be impacted by plaque accumulation and subsequent gingival inflammation [6].

As outlined in the 2017 World Workshop on the Classification of Periodontal and Peri-Implant Diseases and Conditions, drug-influenced gingival enlargements are categorized within dental plaque-induced gingival conditions. The etiology of drug-influenced gingival conditions entails the synergistic action of plaque bacteria and the administered drug to elicit a gingival response. However, not all individuals exposed to these medications exhibit gingival tissue enlargements, indicating a predisposition necessitating specific characteristics [8]. Common clinical features of drug-influenced gingival enlargements include variations in enlargement patterns among individuals, suggestive of a genetic predisposition [9]. These enlargements often manifest with a predilection for involvement of the anterior gingiva [9], with onset typically occurring within 3 months of drug initiation in younger age groups [10]. Initial observation of enlargement frequently occurs in the papillary region [9]. Despite potential occurrence within periodontal tissues with or without bone loss, these enlargements do not correlate with attachment loss or tooth mortality [8]. Notably, clinical and histologic characteristics induced by these drugs demonstrate uniformity, lacking distinguishable differences among them [8].

A significant challenge in managing PV is its complex and variable clinical presentation, which complicates diagnosis and therapeutic interventions. The complexity increases when PV coexists with periodontitis and is further complicated by drug-induced gingival enlargement resulting from PV treatment, which presents additional difficulties for clinicians due to overlapping symptoms and the potential exacerbation of oral lesions. This case report is aimed at elucidating the characteristics of cyclosporin-induced gingival enlargement in a patient with periodontitis and diagnosed with PV.

## 2. Case Presentation

A 44-year-old man was presented to the Periodontics Specialist Clinic, Faculty of Dentistry at the National University of Malaysia (UKM) in 2018 with concerns related to drug-influenced gingival enlargement compounded by underlying PV. Diagnosed with PV in 2018, the patient has been under regular follow-up at the dermatology clinic every 3 months since then. The condition was initially managed with cyclosporin, prescribed at a dose of 100 mg daily, which was later increased to 150 mg in 2022 due to multiple disease flare-ups. Prednisolone was initiated in 2018 and is currently maintained at 5 mg daily, while alendronate (5 mg daily) was prescribed to prevent steroid-induced osteoporosis. In early 2023, cyclosporin was switched to methotrexate (2.5 mg once weekly) as part of a revised treatment plan.

The patient experienced swollen and bleeding gingiva, complicating his daily oral hygiene routine and resulting in persistent swallowing discomfort that affected his dietary habits. He also suffered from multiple episodes of acute exacerbation of chronic oral ulcerations, some requiring hospitalisation in 2018, despite ongoing dermatology follow-up. He continues to attend regular reviews at the oral medicine (OM) clinic every 6 months. Socially, the patient is a medical laboratory technician and a former smoker. His oral hygiene regimen includes brushing twice daily with foaming fluoridated toothpaste and the use of 0.12% chlorhexidine gluconate mouthwash; however, he remains noncompliant with interdental cleaning tools.

**Figure 1:**
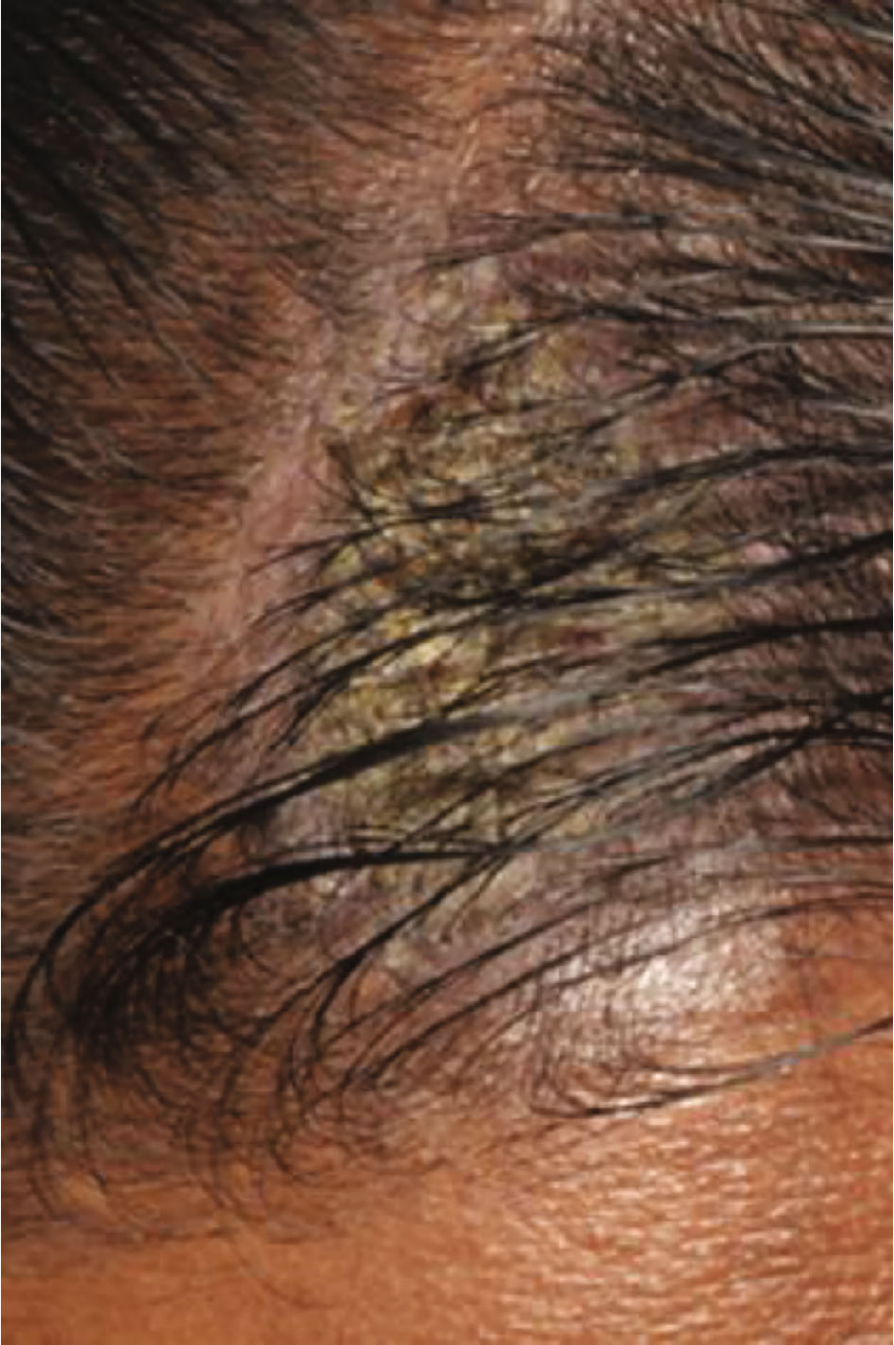
Scab-like patches were noted on the left side of the scalp.

The patient displayed symmetrical facial features, competent resting lips, and a limited mouth opening of approximately two patient's fingers breadth. Scab-like patches were noted on the left side of the scalp (Figure 1), while no bullae lesions were evident on the visible body parts. However, the patient occasionally reported noticing blisters on his back and lower limbs.

**Figure 2:**
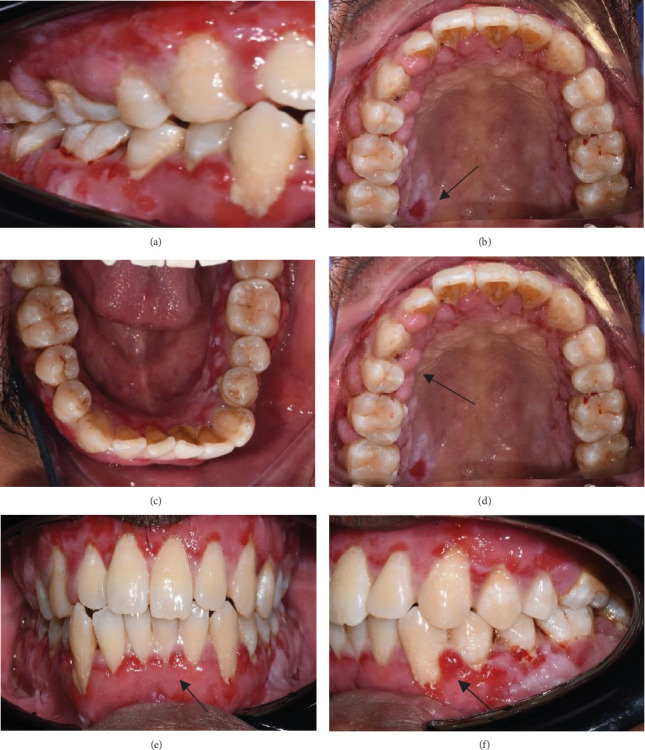
(a) Intraoral photographs show desquamative, whitish, scrapable patches noted on the marginal and attached gingivae extending from 45 to 47 buccally. (b) Isolated, shallow, erythematous raw ulcers were observed on the right hard palate (measuring 2.5 × 2.5 cm). (c) Multiple, shallow, erythematous raw ulcers are present on the lower left lingual. (d) Multiple firm, painless, pale-pink nodular enlargements observed in the interdental papilla extend to the facial and palatal gingival margins in the maxilla. It is delineated by a groove of tissue that does not bleed on touch. (e) Red/bluish-red gingival enlargements (secondary inflammation) were noted at the interdental papilla of the mandibular anterior regions and (f) between Teeth 33 and 34.

Despite the patient's effort to perform good oral hygiene care, the patient exhibited poor oral hygiene, with a full-mouth plaque score of 100% on all surfaces and a full-mouth bleeding score of 37%. The baseline visual analog scale (VAS) for pain was 4 out of 10. Periodontal health assessment revealed generalized oedematous, erythematous marginal gingiva and interdental papillae, with desquamative gingivitis, whitish scrapable patches on the marginal and attached gingivae extending from 45 to 47 buccally and generalized on the lingual aspect on the lower arch (Figure 2(a)). Isolated, shallow, erythematous raw ulcers were observed on the right side of the hard palate, measuring 2.5 × 2.5 cm (Figure 2(b)). Multiple shallow erythematous raw ulcers were also observed on the lower left lingual region (Figure 2(c)). Several painless, firm, pale-pink nodular enlargements were observed in the interdental papilla, localized to the keratinized portions of the gingiva and extending to the facial and palatal gingival margins on the maxilla. The enlargements generally appear more inflamed and exhibit a minimal fibrotic component, more evident in the first and second sextants (Figure 2(d)) than in the rest, which seem to have a smoother texture and are reddish or bluish-red (Figure 2(f)).

**Figure 3:**
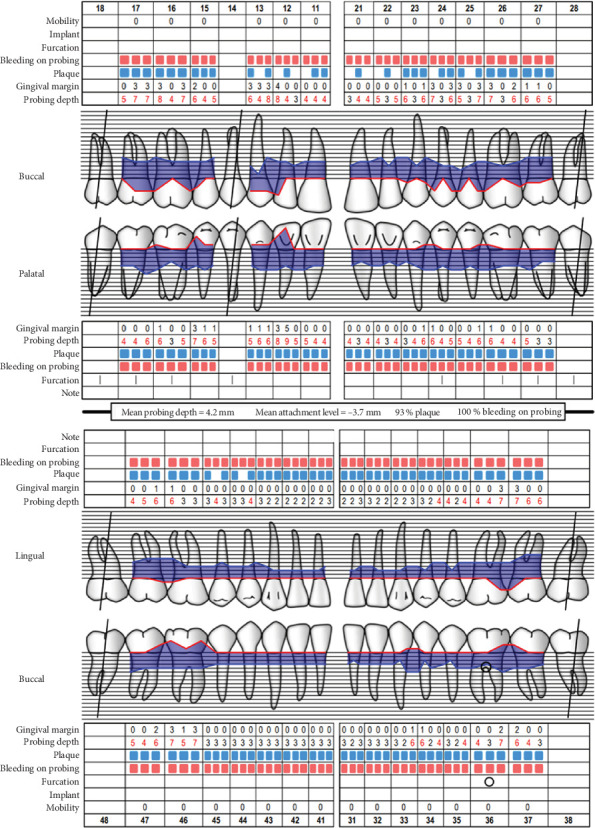
Periodontal health reassessment. *Source:* Department of Periodontology, University of Bern. “Periodontal Chart Online.” Last modified 2010 (http://www.periodontalchart-online.com).

**Figure 4:**
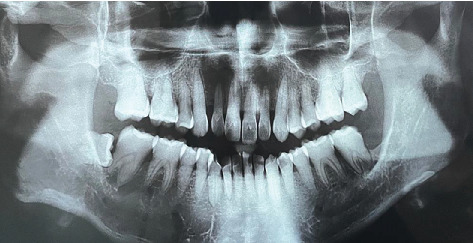
The OPG revealed predominantly horizontal bone loss, ranging from 20% to 30% across most sites. More advanced horizontal bone loss of up to 50% was observed on the distal aspect of Tooth 37, which has a history of surgical removal of an adjacent tooth (MOS). There was also evidence of widening of the periodontal ligament (PDL) space associated with Teeth 36 and 37. Additionally, aberrant root morphology was noted on Tooth 47.

Baseline periodontal charting on 11/04/2022 revealed probing pocket depths ranging from 4 to 9 mm, with bleeding on probing (BOP) recorded at 100% of sites. The full-mouth plaque score was 93%, indicating poor oral hygiene (Figure 3). Additionally, an orthopantomogram (OPG) was taken to evaluate bone levels and detect any underlying alveolar bone loss or other abnormalities, which confirmed generalized moderate horizontal bone loss consistent with the clinical diagnosis of generalized periodontitis (Figure 4).

The patient was diagnosed with generalized gingival enlargement and generalized periodontitis, Stage 3, Grade B, currently unstable with no identifiable risk factors. Nonsurgical periodontal therapy (NSPT) was initiated, and a referral to the OM clinic for the management of PV was made. The treatment plan included multiple periodontal health reassessments and root surface debridement (RSD) sessions performed under local anesthesia. Throughout the course of therapy, comprehensive oral hygiene reinforcement and patient motivation were consistently provided to enhance adherence and optimize clinical outcomes.

The patient's medication regimen includes alendronate 10 mg orally once a day, prescribed since 2018 to prevent steroid-induced osteoporosis. Given the potential risk of osteonecrosis of the jaw (ONJ) associated with bisphosphonate use, precautions were taken to avoid invasive dental procedures. No clinical signs of ONJ have been observed to date. Tailored oral hygiene instructions were provided, particularly to address the patient's immunosuppressive medication regimen, which includes cyclosporin, prednisolone, and later methotrexate. Pharmacological management also included topical corticosteroids and 0.12% chlorhexidine mouthwash twice daily, dexamethasone 0.5 mg (crushed and diluted in water for mouth gargling three times daily), and topical 0.05% clobetasol propionate applied once daily for 2 weeks. Significant improvements in oral hygiene and gingival health were observed by January 2023, 10 months post-treatment.

**Figure 5:**
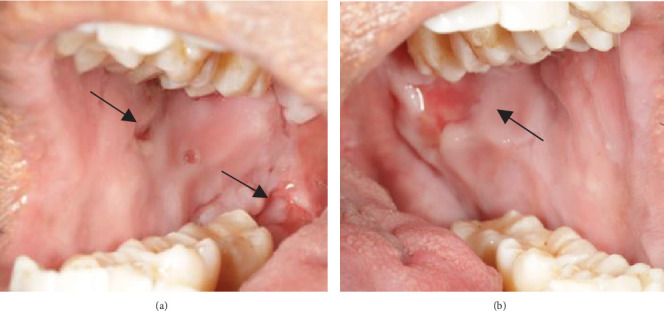
(a, b) Ulcerative lesions present on bilateral buccal mucosa along the level of the occlusal plane and right retromolar pad region.

However, the patient experienced a flare-up of PV in November 2023, resulting in severe pain with a VAS of 8/10 and bilateral ulceration on buccal mucosa at the level of the occlusal plane and right retromolar pad region (Figures 5(a) and 5(b)). The patient was then prescribed systemic corticosteroid in a tapered dosage of prednisolone starting at 10 mg once daily for a week and adjusted to 20 mg once daily for the following 2 weeks when improvement was observed (VAS of 6/10). Following a review at the dermatology clinic, the patient was given an increased dosage of prednisolone, 30 mg, for another 2 weeks. Concurrently, periodontal health management provided at the periodontics clinic focused on oral hygiene modification, advised sodium lauryl sulfate (SLS)–free toothpaste, and prescribed hyaluronic acid mouthwash (Gengigel).

**Figure 6:**
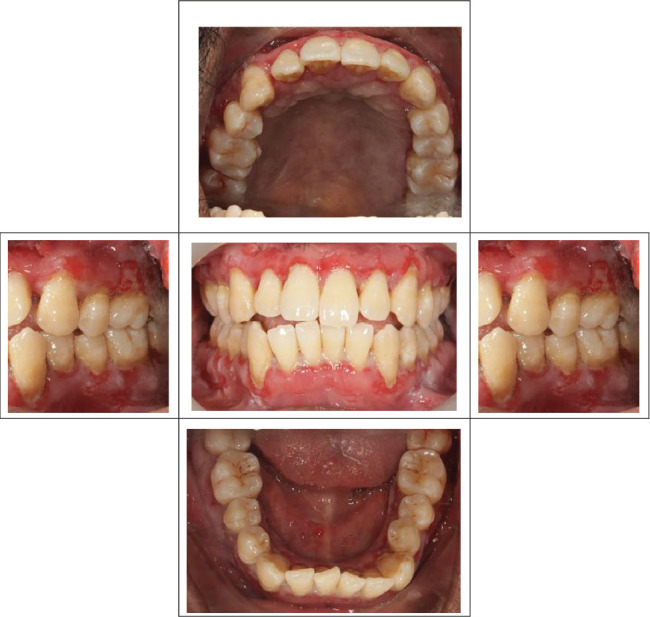
Intraoral clinical photographs depicting the periodontal status after 16 months of discontinuing tab. cyclosporin.

**Figure 7:**
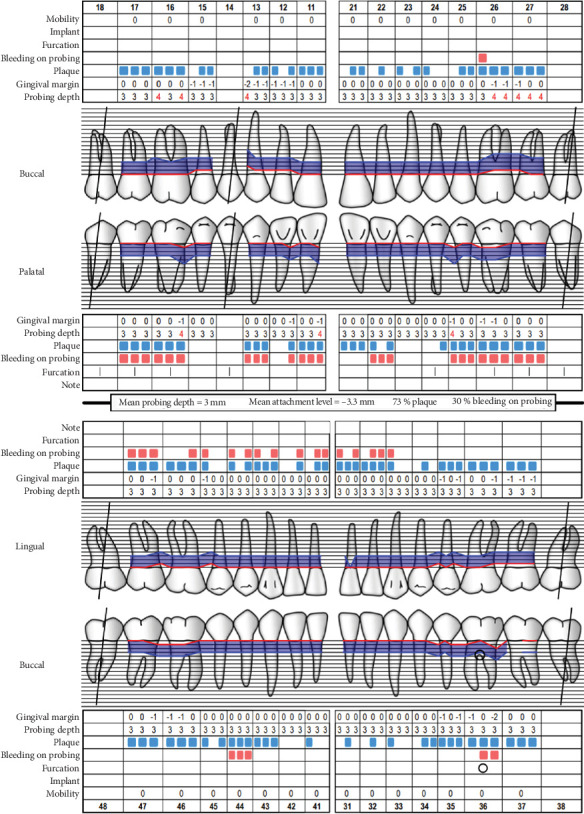
Periodontal health reassessment. *Source:* Department of Periodontology, University of Bern. “Periodontal Chart Online.” Last modified 2010 (http://www.periodontalchart-online.com).

**Figure 8:**
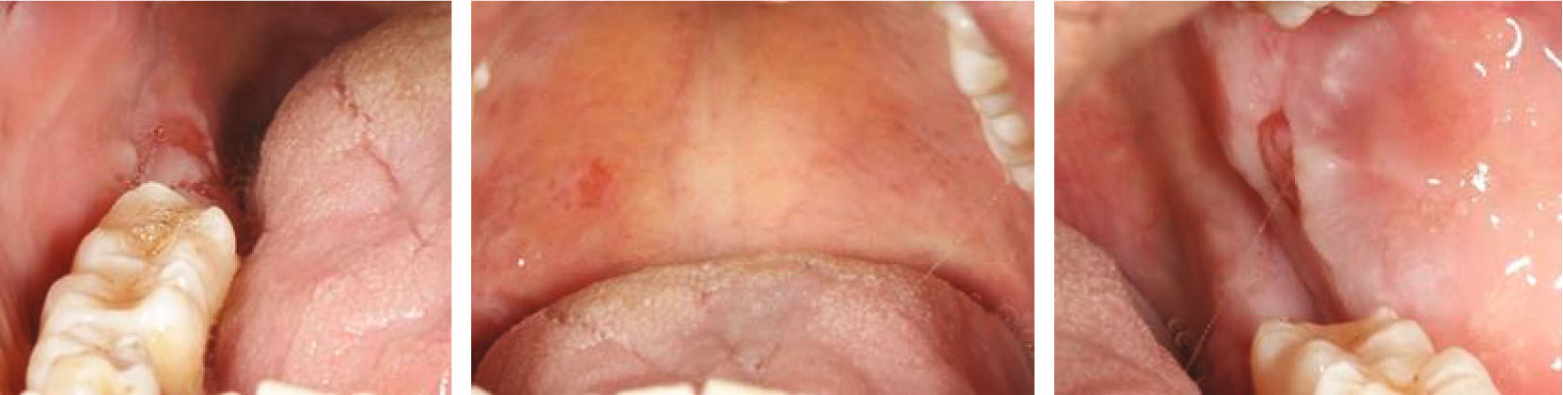
Intraoral photograph demonstrating a reduction in the size of the lesions with partial resolution of the surrounding erythema and inflammation.

At the recent visit in April 2024, the patient reported feeling satisfied with minimal pain (VAS 0–1), and no new oral lesions were observed (Figure 6). Periodontal health reassessment revealed improved oral hygiene status with new and localized plaque primarily on the posterior teeth. Probing pocket depths ranged from 2 to 6 mm, with a marked reduction compared to baseline measurements. BOP had decreased to 31%, and the full-mouth plaque score had improved to 31% (Figure 7). Despite these advancements, erythematous raw ulcers persisted on the right hard palate, the tip of the tongue, bilateral buccal mucosa, and the right retromolar pad region, although the right retromolar pad showed partial resolution of erythema and inflammation. The remaining gingivae were less erythematous and edematous, with complete resolution of gingival enlargements compared to the baseline records (Figure 8). At this stage, the patient was treated with a full-mouth RSD, oral hygiene practices reinforcement, and a prescription of hyaluronic acid mouthwash. Plans for continued NSPT under local anesthesia, oral hygiene education, and follow-up appointments were made to ensure further improvement and maintenance of oral health.

## 3. Discussion

### 3.1. PV: Prevalence, Diagnosis, and Management

The estimated worldwide annual incidence of PV is one to five per 1,000,000 populations [11, 12]. The occurrence is most common in middle-aged and older adults between the fifth and sixth decades of life [12]. Notably, individuals of Jewish or Mediterranean descent exhibit a higher susceptibility to PV compared to other populations, underscoring the significant influence of genetic factors in the disease etiology [13].

The development of intraepithelial bullae in both skin and mucous membranes arises from the production of autoantibodies, specifically IgG, which target desmosome-associated protein antigens, notably desmoglein-3 (Dsg3), localized within the epithelial and epidermal intercellular substance [14–16]. These antigen-antibody complexes disrupt the adhesion function of Dsg3, leading to the detachment of suprabasilar cells (acantholysis) and subsequent blister formation. Upon direct immunofluorescence examination, antibodies directed against Dsg3 were seen as deposits along the periphery of epithelial cells, often resembling a “chicken wire” pattern [14]. Additionally, circulating IgG targeting Dsg3 can be identified in serum samples through indirect immunofluorescence assay.

PV typically presents with extensive bulla formation, often affecting large areas of the skin, and if left unaddressed, the condition poses a life-threatening risk [17, 18]. Gingival involvement, characterized by desquamative gingivitis and/or the presence of vesiculobullous lesions on both free and attached gingiva, featuring intraepithelial bullae that, upon rupture, result in denuded and haemorrhagic erosions [17, 18], is common. The oral mucosa is frequently affected, with approximately 54% of cases reporting the oral cavity as the primary site of involvement [19]. Clinically, lesions present as fragile blisters observed throughout the oral cavity, particularly in regions subjected to frictional trauma, with the buccal mucosa being the most commonly affected site, followed by the palate and tongue [20]. Since oral manifestations may precede systemic involvement, dental practitioners must promptly recognize these presentations and facilitate appropriate referrals. Early diagnosis and treatment of oral lesions are paramount, as untreated generalized PV can lead to fatal outcomes, underscoring the importance of timely intervention to optimize prognostic outcomes.

The diagnosis of PV relies on clinical presentation, characterized by the fragility of the oral mucosa, often eliciting Nikolsky's sign, wherein gentle pressure leads to the separation of epithelial layers, leaving behind a yellowish-white pseudomembrane on the affected area that subsequently peels off, revealing an underlying erythematous surface prone to bleeding upon palpation. Confirmation of diagnosis typically involves histopathological examination, which reveals histologic features consistent with intraepithelial bulla formation resulting from desmosome destruction and subsequent acantholysis. Additionally, circulating autoantibody titers targeting Dsg1 and Dsg3 can be detected using enzyme-linked immunosorbent assay, providing further diagnostic support [6]. Histologically, the bullae exhibit nonadhering free epithelial cells, known as Tzanck cells, which lack intercellular bridges due to desmosomal disruption [21, 22]. Infiltration of mononuclear cells and neutrophils characterizes the associated inflammatory reaction.

Dental practitioners may encounter challenges in accurately diagnosing and managing oral pemphigus, potentially leading to misdiagnosis and inappropriate treatment modalities. Differential diagnoses commonly considered include recurrent aphthous ulceration, Behçet disease, erosive lichen planus, oral candidiasis, and erythema multiforme [23]. Discriminating among these conditions necessitates a meticulous assessment of patient history and thorough clinical examination. For instance, erythema multiforme often presents with target-shaped skin lesions and lip involvement, distinguishing it from other conditions. Erosive lichen planus is typified by Wickham's striae and erosions, particularly in its erosive form. In paediatric populations, differential diagnosis of oral pemphigus encompasses acute herpetic gingivostomatitis, impetigo, linear IgA disease, and epidermolysis bullosa. Moreover, mucous membrane pemphigoid emerges as a pertinent differential diagnosis for PV, particularly among individuals aged over 60 years. Its clinical presentation typically includes recurrent episodes of tense subepithelial bullae, predominantly affecting multiple mucosal sites, occasionally involving the skin [23].

A pivotal aspect of managing PV is early diagnosis, enabling the administration of low-dose medications for shorter durations to control the disease and prevent potentially fatal complications such as loss of the epidermal barrier leading to dehydration and secondary bacterial infection [24]. Typically, PV is managed using topical and systemic steroids, with guidelines advocating a two-phase approach: an induction phase for disease control and a maintenance phase for treatment tapering [25]. Local treatments like ointments and mouthwashes can manage symptoms, particularly in patients with low circulating autoantibody titers, utilizing agents such as 0.1% triamcinolone acetonide in Orabase, 0.05% clobetasol propionate, or 0.05% halobetasol [20, 23]. For refractory lesions, intralesional injections of triamcinolone acetonide or paramethasone can be administered, though discontinuation should be considered if symptoms persist [20, 23]. Systemic corticosteroids are promptly initiated in patients with extensive oral ulceration or cutaneous involvement, with a recommended prednisolone dose gradually reduced to minimize side effects. Steroid-sparing drugs like cyclophosphamide and azathioprine may be added to the regimen if long-term steroid use is necessary to manage complications [25].

Additionally, general dental practitioners may contribute to patient well-being by maintaining strict oral hygiene, providing periodontal treatment, offering dietary advice, monitoring prosthetic restorations, and administering anticandida medications for patients on long-term steroids [20, 26]. Recurrences are common due to poor patient cooperation and poor oral hygiene. Also, the use of dental prostheses and dental restorations, oral habits, smoking, and alcohol consumption can complicate the management [7, 27, 28].

### 3.2. Cyclosporin-Influenced Gingival Enlargement

The reported incidence of cyclosporin-influenced gingival enlargement spans a wide range from 3% to 100%. However, in relatively well-controlled studies, the overall prevalence appears to be approximately 25%, irrespective of whether cyclosporin was administered for graft rejection prevention or for managing other autoimmune disorders [26, 29]. Young individuals tend to exhibit a higher incidence of cyclosporin-induced gingival overgrowth than older individuals [30, 31]. Daley and Wysocki [30], in a study of 100 subjects under a chronic cyclosporin regimen, found no direct correlation between the oral dose of cyclosporin and the severity of gingival overgrowth. Conversely, Phillips et al. [32] reported no correlation between cyclosporin dose and the presence of overgrowth in their evaluation of 50 transplant patients.

Schulz et al. [33] observed no significant correlation between plaque score, gingival inflammation severity, and gingival overgrowth. Tyldesley and Rotter's [34] survey suggested that overgrowth severity might be worse in subjects with poor plaque control, although overgrowth commonly occurred in patients with optimum oral hygiene. Hefti et al. [35] indicated that even with maximum oral hygiene, approximately 22% of cyclosporin patients would exhibit gingival overgrowth, indicating that prevalence is not linked to oral hygiene or gingival inflammation. Therefore, it is reasonable to propose that proper oral hygiene could minimize the severity of cyclosporin-induced gingival enlargement by eliminating the inflammatory component. Animal studies have shown that gum lesions develop rapidly (within 3–6 weeks of initiating a daily cyclosporin regimen) and regress spontaneously upon withdrawal of cyclosporin [36]. Seibel et al. [36] conducted a well-designed study using the Beagle model to investigate oral hygiene's effect on cyclosporin-induced gingival overgrowth. They demonstrated that a rigorous daily toothbrushing regimen, implemented after developing cyclosporin-induced gingival lesions, effectively reduced clinical manifestations of gingival inflammation but had no impact on reducing gingival mass.

Microscopic observation reveals that most cases of gingival enlargement are characterized by an apparent augmentation in the collagenous elements within the connective tissue matrix [10, 30, 34, 37]. However, a definitive increase in fibroblasts (true hyperplasia) was not casually observed [38]. Deliliers et al. [37] asserted that the redundancy of oral tissues resulted solely from epithelial acanthosis and the accumulation of extracellular substances, often referred to as “ground substances.”

## 4. Concluding Remarks

In individuals diagnosed with drug-influenced gingival enlargement, periodontitis, and PV, the interplay among these conditions can markedly impact both systemic as well as oral health. Therefore, meticulous and periodic monitoring, coupled with comprehensive periodontal intervention, is crucial for effectively managing symptoms associated with pemphigus flare-ups and the potential drug side effects. Drug-influenced gingival enlargement has a good prognosis and is generally reversible on stopping or substituting the offending drug. This comprehensive strategy focuses on symptom alleviation and enhances the individual's overall well-being and oral health-related quality of life. This case highlights the significance of collaborative care among various dental and medical disciplines to address the patient's signs and symptoms adequately.

## 5. The Takeaways

PV represents a severe autoimmune disorder with the potential for life-threatening complications affecting both the skin and mucous membranes. Oral lesions commonly serve as the initial clinical presentation in most cases, underscoring the significance of promptly recognizing these manifestations. Gingival enlargements influenced by cyclosporin may potentially have adverse outcomes when treating PV. Hence, clinicians must emphasize enough on patient education, particularly on the potential risk of gingival enlargement before starting treatment. Emphasizing the importance of maintaining effective oral hygiene is also essential to mitigate these risks. Dental professionals play a critical role in the early detection and management of PV and the side effects of its drug intervention by possessing a comprehensive understanding of its clinical features, thereby influencing the disease prognosis. Accurate clinical assessment, complemented by adjunctive diagnostic measures such as routine biopsy and immunofluorescence studies, is imperative in distinguishing PV from other vesiculobullous lesions and facilitating appropriate therapeutic interventions.
